# Feature Extraction and Selection for Myoelectric Control Based on Wearable EMG Sensors

**DOI:** 10.3390/s18051615

**Published:** 2018-05-18

**Authors:** Angkoon Phinyomark, Rami N. Khushaba, Erik Scheme

**Affiliations:** 1 Institute of Biomedical Engineering, University of New Brunswick, Fredericton, NB E3B 5A3, Canada; escheme@unb.ca; 2 Faculty of Engineering and Information Technology, University of Technology, Sydney 2007, NSW, Australia; rami.khushaba@uts.edu.au

**Keywords:** electromyography, EMG, feature extraction, L-moments, pattern recognition, prosthesis, sampling rate, wearable sensor

## Abstract

Specialized myoelectric sensors have been used in prosthetics for decades, but, with recent advancements in wearable sensors, wireless communication and embedded technologies, wearable electromyographic (EMG) armbands are now commercially available for the general public. Due to physical, processing, and cost constraints, however, these armbands typically sample EMG signals at a lower frequency (e.g., 200 Hz for the Myo armband) than their clinical counterparts. It remains unclear whether existing EMG feature extraction methods, which largely evolved based on EMG signals sampled at 1000 Hz or above, are still effective for use with these emerging lower-bandwidth systems. In this study, the effects of sampling rate (low: 200 Hz vs. high: 1000 Hz) on the classification of hand and finger movements were evaluated for twenty-six different individual features and eight sets of multiple features using a variety of datasets comprised of both able-bodied and amputee subjects. The results show that, on average, classification accuracies drop significantly (p< 0.05) from 2% to 56% depending on the evaluated features when using the lower sampling rate, and especially for transradial amputee subjects. Importantly, for these subjects, no number of existing features can be combined to compensate for this loss in higher-frequency content. From these results, we identify two new sets of recommended EMG features (along with a novel feature, L-scale) that provide better performance for these emerging low-sampling rate systems.

## 1. Introduction

As many amputees and individuals with impaired motor function have difficulty using traditional user interfaces (e.g., joysticks, mice, and keyboards) or assistive and rehabilitative devices, more advanced hands-free human–computer interfaces are desirable. Recognition of human muscle activity, facilitated using surface electromyographic (EMG) signals generated during muscular contractions, has been seen as one of the more promising solutions [1,2]. To create EMG-based human–computer interfaces to be used in an everyday context, they should be simple and non-invasive, such as a watch, an armband, jewelry, or concealed beneath clothing [3]. With the advances in wearable sensors, wireless communication and embedded computing technologies, we can indeed now obtain EMG data unintrusively using wearable EMG armbands (for a review, see [4]). These EMG armbands typically include multiple EMG sensors positioned radially around the circumference of a flexible band, allowing ease of donning and wear in daily life. Arguably the most widely known EMG armband is the Myo armband by Thalmic Labs, a low-cost consumer-grade EMG device integrating an ARM Cortex-M4 based microcontroller unit, a set of eight dry EMG electrodes, a nine-axis inertial measurement unit (IMU) and a Bluetooth Low Energy (BLE) module. These EMG armbands have unlocked new possibilities for myoelectric control applications, which are not limited to the more traditional prosthetics market. Applications have included accessible game-based training for myoelectric prostheses [5,6], sport training systems [7], portable music player interfaces [3], sign language interpretation [8], and user authentication [9].

Pattern recognition techniques have long been proposed as a way of extracting considerable information from EMG sensors for recognition of human activity [1,2], and have the potential to restore more degrees of freedom than conventional control techniques. Using pattern recognition approaches, it is not necessary to measure EMG signals only from independent muscles, enabling physiologically appropriate control [10].

These techniques therefore offer a logical extension for newer wearable systems. EMG pattern recognition systems generally consist of four inter-connected components: data pre-processing, data windowing, feature extraction, and classification [11]. Specifically, the raw EMG signals are pre-processed and segmented into a series of overlapping windows of finite length. From these windows, a set of relevant metrics, called features, are extracted from each window, effectively projecting the signals to a lower dimensional space and increasing their information density. A classifier then recognizes signal patterns and discriminates them into pre-defined classes. Because the extracted features represent the EMG signal characteristics for classification, many previous studies have shown that the success of pattern recognition-based myoelectric control depends almost entirely on the extraction and selection of high-quality and representative features [12,13,14]. Due to physical, processing, data transmission and power consumption constraints, however, wearable EMG armbands typically sample EMG signals at a lower frequency than is done clinically (e.g., 200 Hz for the Myo armband). As it is widely known that the spectra of EMG signals are distributed within a range of 20–500 Hz, it remains unclear whether existing EMG feature extraction methods [12,13,14], which have been developed based on sampling EMG signals at or above the Nyquist rate (usually 1000 Hz), are still effective for use with low-resolution sensor systems.

Several studies have investigated the effects of sampling rate on classification accuracy of hand and finger movement tasks down-sampling higher-frequency EMG recordings, but with mixed results [15,16,17,18]. Three studies reported that the classification accuracies obtained from a set of multiple time domain features dramatically decrease when the sampling rate is lower than 200 Hz [15], 400 Hz [16], or 600 Hz [17]. In contrast, the other study [18] reported that reducing the sampling rate does not degrade the classification performance of the similar feature set. This study also examined the performance of myoelectric control as the resolution of analog to digital (A/D) conversion is decreased, and suggested that the resolution of the A/D conversion can be reduced to 8-bit while maintaining or even improving classification performance [18]. Thus, with the growing adoption of lower-bandwidth sensor systems, a better investigation of how the classification performance of EMG feature extraction methods is affected by lower sampling rates is warranted.

Most recently, some studies have collected EMG data directly from a wearable EMG armband at a low sampling rate (usually the Myo armband), and evaluated the performance of EMG feature extraction methods [19,20,21]. These studies, however, are limited in terms of the relatively small sample sizes used for classification (e.g., only a single subject [19]) and/or the small number of EMG features used for comparison (e.g., 1 [20,21], 5 [19], and 6 [22] features). Furthermore, these aforementioned studies did not investigate the effects of other factors that have been shown to greatly influence the performance of EMG pattern recognition systems, such as the changing characteristics of the EMG signal itself over time [23,24], change in limb position [25,26], change in forearm orientation [27], and variation in muscle contraction effort [27,28]. Consideration of these confounding factors is essential in order to make myoelectric control practical and robust in a real-world context.

The first purpose of this study was therefore to examine the effects of sampling rate (low: 200 Hz vs. high: 1000 Hz) on classifying basic and functional movements of the hand and fingers in both able-bodied and amputee subjects for a broad range of individual and multi-feature sets. The 200 Hz frequency was chosen exclusively for comparison as the sampling rate used by the predominant commercially available consumer-grade wireless myoelectric armband. The second purpose of this study was to identify a set of features that is more accurate and robust for these new systems.

The current study extends the prior literature by considering 26 individual commonly used and newly proposed features, as well as eight different state-of-the-art multi-feature sets. Since reducing sampling rate decreases the number of data points, several large outlying data values (e.g., spurious background spikes) could highly influence features computed using conventional measures (e.g., mean, standard deviation, and variance) for an analysis window with a small number of data points [29]. Therefore, in this study, we also proposed to use a robust measure of statistical dispersion based on L-moment [30], which is unaffected by small numbers of outliers. Furthermore, four datasets from previous studies were used to investigate the confounding effects of sampling rate and practical robustness issues: (1) change in *limb position*; (2) change in *forearm orientation*; (3) variation in *contraction intensity*; and (4) amputee or transradial *amputation*.

Ultimately, this study highlights the differences in efficacy of EMG features as a result of lower sampling rates found in emerging wearable EMG technology, and informs the design and selection of correspondingly resilient features.

## 2. Materials and Methods

### 2.1. EMG Datasets

Surface EMG data obtained from four different datasets comprised of forty subjects (31 able-bodied subjects and 9 transradial amputees) were analyzed in this study. These datasets were collected independently at different institutes and are publicly available [26,27,28,31]. Because performance of EMG features can vary depending on experimental differences between datasets, multiple datasets are advantageous when examining the robustness and generalization of research findings [32,33]. The first three datasets were collected using high-resolution EMG recording systems and were used in this study to investigate practical robustness issues, while the last EMG dataset was collected using the Myo armband and was used in this study to identify a set of recommended EMG features for low sampling rate myoelectric control systems.

The experiment from which the last EMG dataset resulted was divided into three exercises [34]. Exercise A consists of 12 basic movements of the fingers (flexions and extensions). Exercise B consists of 8 isometric and isotonic hand configurations and 9 basic movements of the wrist (adduction/abduction, flexion/extension, and pronation/supination). Exercise C consists of 23 grasping and functional movements. It should be noted that all evaluated datasets were collected with an EMG armband placed around the circumference of the subject’s forearm. More details about subjects, experiments, and data acquisition are reported in Table 1.

Prior to analysis, EMG data were notch (50 Hz) and band-pass (20–500 Hz) filtered using a digital Butterworth finite impulse response (FIR) filter of order 4 to remove any power-line interference, motion artifact, and high-frequency random noise, which could possibly affect the performance of EMG features [35,36]. Data analyses in this study were conducted in accordance with the University of New Brunswick Research Ethics Review Board (REB 2008-083) while the ethics approvals and consent to participate for each dataset can be found in the original works [26,27,28,31].

### 2.2. Feature Extraction

Twenty-six feature extraction methods in time domain (24) and frequency domain (2) were selected from four functional EMG feature groups: the signal amplitude and power feature group, the nonlinear complexity and frequency information feature group, the time-series modeling feature group, and the unique feature group, to cover all types of meaningful information for EMG signal classification [32]. All feature extraction methods were performed using a window size of 250 ms with an increment of 125 ms (50% overlap), which has been shown to be suitable for use in real-time on an embedded system [37].

#### 2.2.1. The Signal Amplitude and Power Feature Group

Eleven features were selected from the first functional feature group, which is used to estimate signal magnitude and power. These features are comprised of six commonly used features: integrated absolute value (IAV), mean absolute value (MAV), root mean square (RMS), variance (VAR), waveform length (WL), and log detector (LD) [12]; four recently proposed features: difference absolute mean value (DAMV), difference absolute standard deviation value (DASDV), difference variance value (DVARV), and the mean value of the square root (MSR) [38,39]; and a newly proposed feature in the present study, referred to as L-scale (LS). This novel feature for EMG is far less sensitive to outliers as compared to standard deviation because it uses the concept of L-moments (a linear function of the expected order statistics) [30]. If *X* is a real-valued random variable, the *r*th L-moment of *X* can be defined as

(1) λr=r−1∑k=0r−1(−1)kr−1kEXr−k:r,

where *r* is set at 2, $X_{k:n}$ denotes the *k*th order statistic of a random sample of size *n*, and *E* denotes the expected value. For an extended coverage of the LS and L-moment, the reader is encouraged to consult the original work [30].

#### 2.2.2. The Nonlinear Complexity and Frequency Information Feature Group

The second functional feature group can be divided into two sub-groups: three nonlinear and complex features and five frequency information features. The first sub-group consists of maximum fractal length (MFL), detrended fluctuation analysis (DFA) and sample entropy (SampEn) [24,40]; and the second sub-group comprises zero crossing (ZC), slope sign change (SSC), Willison amplitude (WAMP), median frequency (MDF), and mean frequency (MNF) [12].

#### 2.2.3. The Time-Series Modelling Feature Group

The third functional feature group includes the coefficients of time-varying linear predictive models: autoregressive coefficients (AR) and cepstrum coefficients (CC) [12]. Three different orders, namely 4, 6, and 9, of these features were employed [41,42,43]. It should be noted that these feature extraction techniques provided more than one feature value, and CC was derived from the AR model [43].

#### 2.2.4. The Unique Feature Group

This last functional feature group was named unique, as it contains many varying feature extraction techniques [32]. Although features in this group are unique, capturing different kinds of information from the EMG signals, most of the features in this group could be considered as an extension of features in other groups and the individual discriminant power of features in this group is lower than features in other groups [32]. However, in this study, histogram (HIST) with nine data bins was chosen as a representative feature for this group based on its popularity in the literature [14]. It should be noted that HIST could be considered as an extension of the ZC and WAMP features.

#### 2.2.5. Multi-Feature Sets

In addition to the individual features, eight different, previously proposed, multi-feature sets were evaluated in this study:

- *MS1 (Hudgins’ set of TD features)*: MAV, WL, ZC, and SSC [44]
- *MS2*: RMS and AR6 [42]
- *MS3*: MAV, WL, ZC, SSC, RMS, and AR6 [42]
- *MS4*: AR4 and HIST9 [41]
- *MS5*: WL, LD, SSC, and AR9 [43]
- *MS6*: WL, SSC, AR9, and CC9 [43]
- *MS7*: RMS, VAR, LD, and HIST9 [43]
- *MS8*: WL, RMS, SampEn, and CC4 [24]

### 2.3. Feature Evaluation and Selection

For comparison, EMG signals from the first three datasets (Datasets 1–3) were down-sampled from the original sampling rate (Table 1) to 1000 Hz and 200 Hz using an anti-aliasing FIR low-pass filter. The dependence of the classification performance on the reduced sampling rate was investigated using a 10-fold cross-validation classification rate obtained from a support vector machine (SVM) classifier with a linear kernel [45]. Specifically, all data were randomly partitioned into 10 equally sized sub-datasets and a single sub-dataset was retained as testing data while the remaining 9 sub-datasets were used as training data for the classification model. The cross-validation process was then repeated for each of the 10 sub-datasets, and a single classification rate was computed by averaging from 10 results. For a common dataset, higher classification rates imply a higher degree of class separability or improved repeatability, both of which are desirable. The classifiers were also trained and tested independently on data from each subject. It should be noted that SVMs were employed because, in the literature, this classifier has shown a better classification performance than other commonly used classifiers such as linear discriminant analysis (LDA) and artificial neural networks [34,46,47]. In previous work [48], SVMs were tested and provided similar trends as other quantitative measures of feature space quality involving LDA, Davies–Bouldin index, and separability index [14,49]. This classification approach was also used to evaluate the classification performance of the EMG features for myoelectric control based on wearable EMG sensors (Dataset 4).

Paired-sample *t*-tests were used to test for differences between means of classification rates and results were considered significant for p<0.05. The resulting *p*-values were adjusted using a Holm–Bonferroni method to maintain a family-wise alpha of 0.05 for tests on all single features and multi-feature sets. The size (or meaningfulness) of differences observed was measured using Cohen’s effect size, *d*, defined as the difference between two group means divided by a standard deviation [50]. For interpretation and consistency, an effect size of 0.2 equates to a small effect, 0.5 equates to a medium effect, 0.8 equates to a large effect, 1.2 equates to a very large effect, and larger than 2.0 equates to a huge effect [50,51].

To examine whether the rank of features used to create a subset of multi-features that best predict the motion classes is the same for the two different sampling rates, a sequential forward selection (SFS) algorithm was performed independently for each case (Dataset 3) [52,53]. This feature selection approach was also used to identify a set of recommended EMG features that provide better performance for low-sampling rate systems (Dataset 4). EMG feature sets were selected using 70% of the data (a training set) and the performance of the selected features was examined using the remaining (30%) of the data (the testing set). The SFS method was applied across subjects using the 10-fold cross-validation method of within-subject classification rates on the training set.

## 3. Results

**Figure 1:**
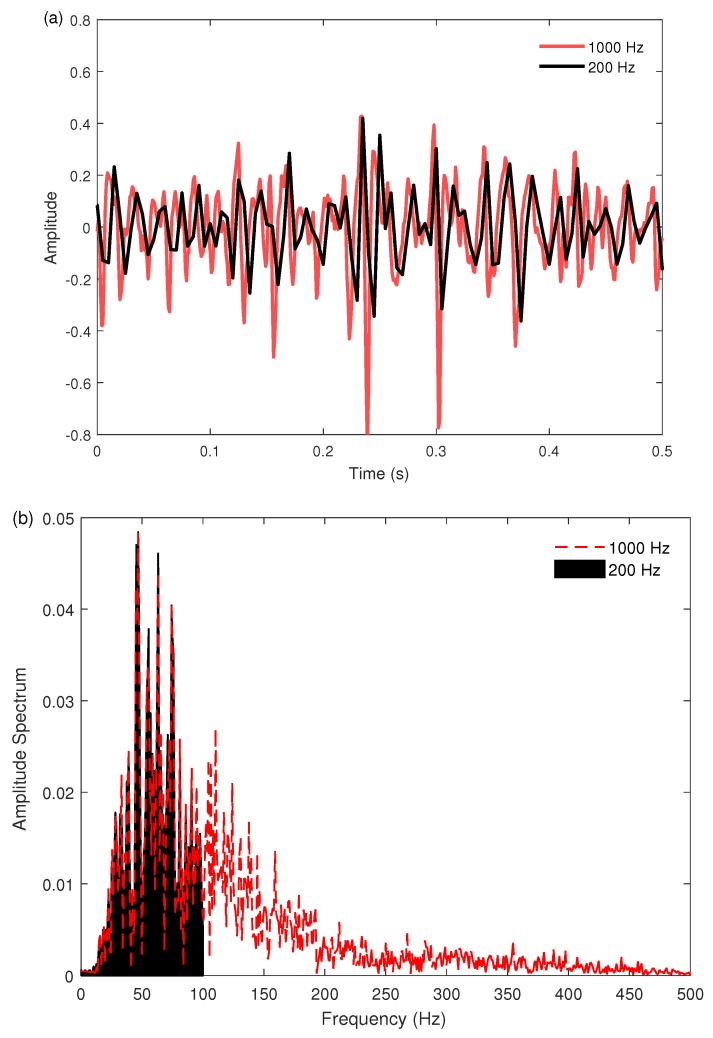
Examples of EMG data sampled at two different sampling rates (1000 Hz vs. 200 Hz) in: (**a**) time domain; and (**b**) frequency domain. Samples are acquired from the first EMG channel of Subject 1 during the hook grip motion from Database 3.

For the first aim of this study, the classification performance of the eight multi-feature sets and twenty-six single features was examined for the two sampling rates using an SVM classifier with 10-fold cross validation. Classifiers were trained and tested using EMG features from all conditions for each of the practical issues, and the results are shown in Table 2 and Table 3, respectively. In addition to the quantitative measures, visual inspection of the graphs was performed. Figure 1 shows a comparison of surface EMG signals when down-sampled from 1000 Hz to 200 Hz in both time- and frequency-domains.

According to the Nyquist theorem, the sampling rate must be at least twice the highest frequency of interest in a signal, and thus the highest frequency component correctly represented for EMG signals sampled at 200 Hz and 1000 Hz is 100 Hz and 500 Hz, respectively.

**Figure 2:**
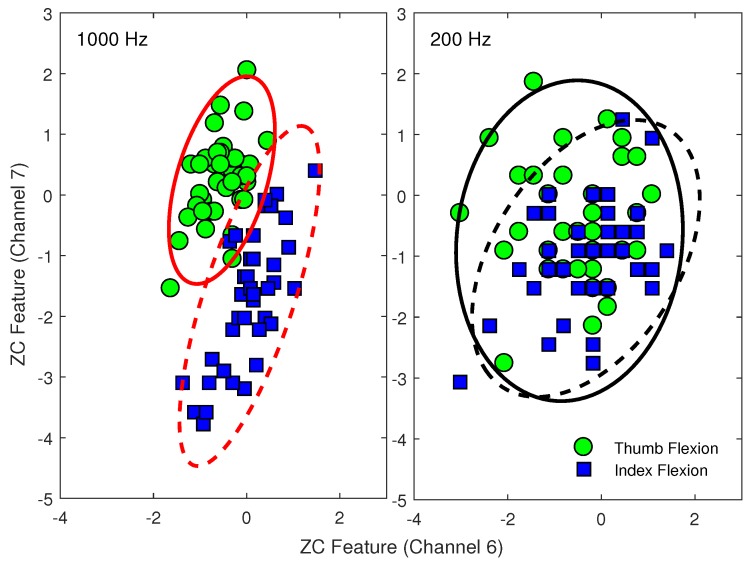
Differences in EMG patterns between using: (**left**) a 1000 Hz sampling rate; and (**right**) a 200 Hz sampling rate. ZC features are extracted from two different EMG channels (6 and 7) during thumb flexion (green circle markers and solid lines) and index flexion (blue square markers and dashed lines). Samples are from Subject 1 of Database 3.

The substantial loss of relevant signal power is visible in each domain. As a further illustration of the changes of EMG feature patterns due to down-sampling, representative scatter plots of the ZC features extracted from two different EMG channels of an amputee subject are shown in Figure 2.

**Figure 3:**
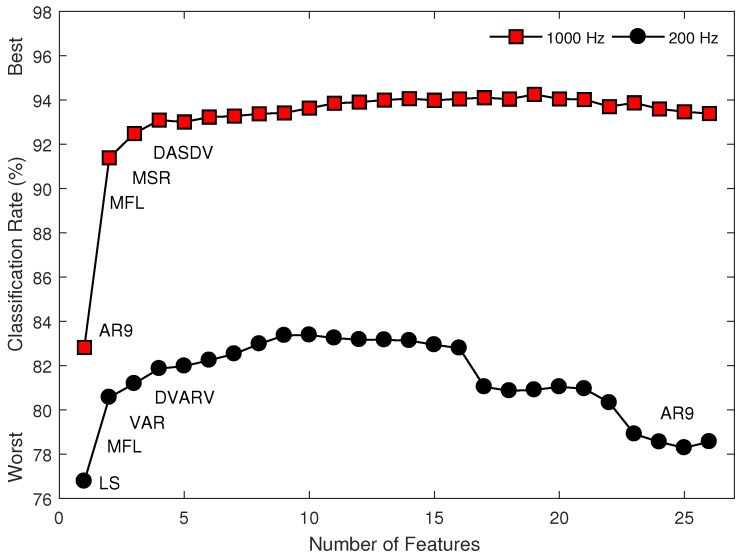
The classification performance of the multi-feature sets sequentially selected by the SFS method using the two different sampling rates (1000 Hz vs. 200 Hz) for Dataset 3.

**Figure 4:**
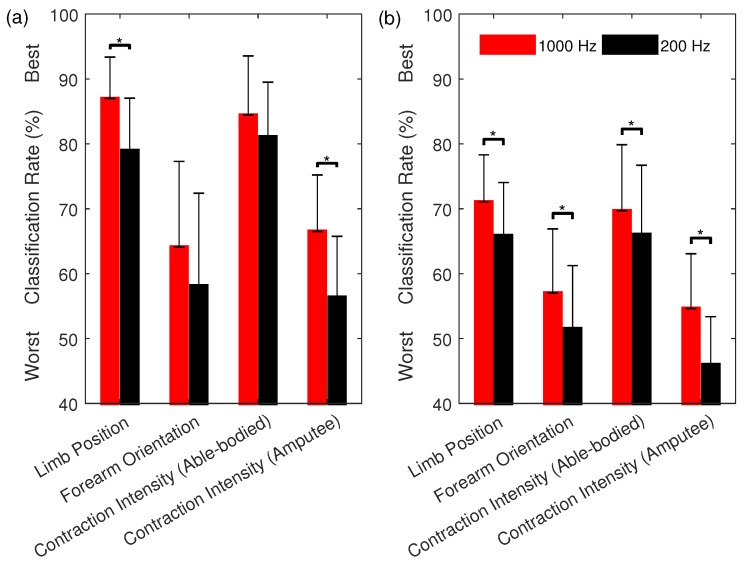
The classification performance of the MS1 feature set using an SVM classifier for multiple EMG datasets (Datasets 1–3) when: (**a**) the training set is acquired from multiple conditions and the testing set is acquired from a different condition, unseen during training; and (**b**) the training set is acquired from a single condition and the testing set is acquired from multiple conditions. Mean values across subjects and classes of motion represented with bars and their standard deviations with error bars. Asterisks indicate significant difference (p<0.05).

The rank and classification performance of the multi-feature sets, as selected using SFS (from sets of one to twenty-six features), are shown in Figure 3. To illustrate the effect of training strategies, Figure 4 shows the classification performance of the most commonly used multi-feature set, Hudgins’ TD (MS1), under two different training strategies. The first strategy is to train a classifier with the EMG features from all the conditions, except the one being tested on. The second strategy is to train a classifier with the EMG features from each of the conditions individually while testing the trained classifier on unseen data from all possible conditions.

**Figure 5:**
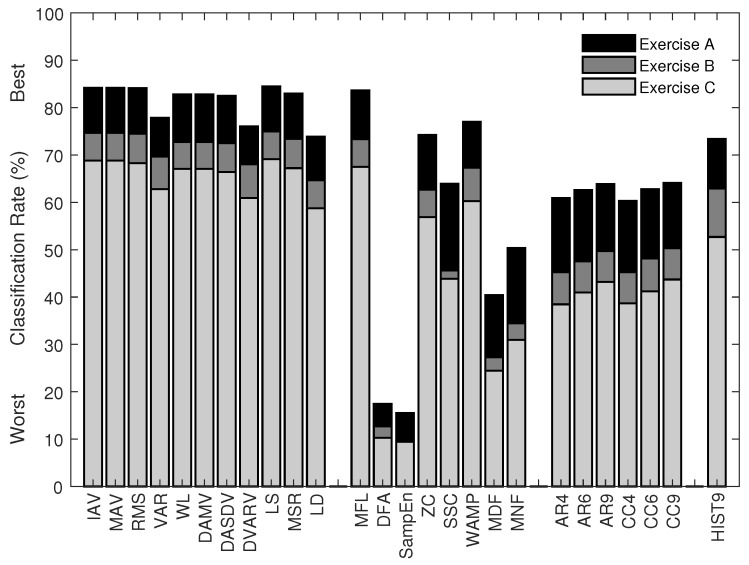
The classification performance of 26 individual features of 12 motion classes in Exercise A, 17 motion classes in Exercise B, and 23 motion classes in Exercise C using SVM classification of myoelectric signals recorded from wearable EMG sensors from Dataset 4 (with a sampling rate of 200 Hz).

**Figure 6:**
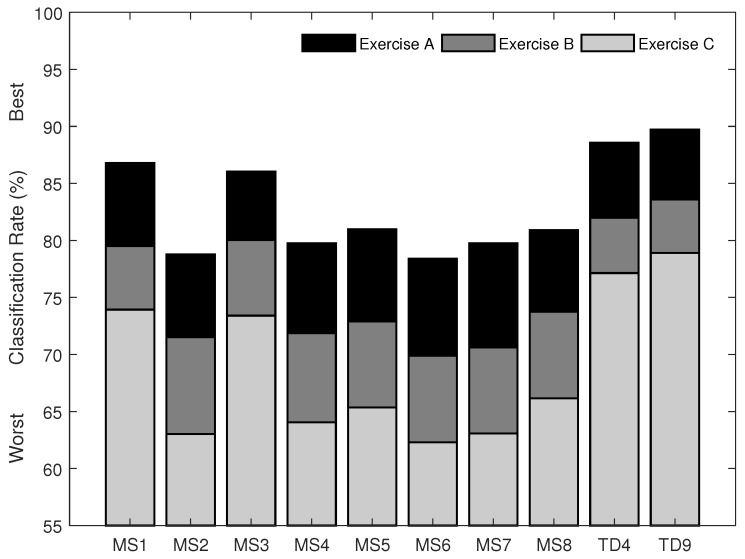
The classification performance of eight multi-feature sets (MS1–MS8) and two newly proposed multi-feature sets (TD4 and TD9) in the classification of 12 motion classes in Exercise A, 17 motion classes in Exercise B, and 23 motion classes in Exercise C using SVM classification of myoelectric signals recorded from wearable EMG sensors from Dataset 4 (with a sampling rate of 200 Hz).

For the second aim of this study, the classification performance of the twenty-six single features, the eight previously proposed, and two newly proposed multi-feature sets was examined using EMG data recorded from the Myo armband, and the results are shown in Figure 5 and Figure 6, respectively. The newly proposed multi-feature sets were determined using the SFS method. The first chosen by selecting the first local maximum after which no meaningful improvement was found (d<0.2). This occurred including between four and five features, and resulted in classification accuracies of 88.6% for Experiment A, 82.0% for Experiment B, and 77.1% for Experiment C, when four features were used. The chosen features are *LS, MFL, MSR, and WAMP*, and are referred to here as *the new set of four time domain features* (*TD4*). The second was chosen as the set that obtained the highest overall classification rates (89.7% for Experiment A, 83.6% for Experiment B, and 78.9% for Experiment C) and consisted of nine features, namely *LS, MFL, MSR, WAMP, ZC, RMS, IAV, DASDV, and VAR*, referred to here as *the new set of nine time domain features (TD9)*.

## 4. Discussion

The first aim of this study was to examine the influence of two different common sampling rates (low: 200 Hz vs. high: 1000 Hz) on the classification of different hand and finger movements in able-bodied and amputee subjects. The present results show that the classification performance of all eight of the tested state-of-the-art multi-feature sets dropped significantly (p<0.05) for all datasets and known practical issues (Table 2) when dropping the sampling rate from 1000 Hz to 200 Hz. These findings suggest that the lower sampling rate could not preserve sufficient control information for accurate classification of six-to-seven classes of hand and finger motions using 6–8 EMG channels. Although the dominant energy of surface EMG signals is in the range of 50–150 Hz, it is reaffirmed here that important signal energy exists at higher frequencies. It is clearly seen here that the accuracy in identifying multiple classes of hand and finger motions is shown to benefit from these high frequency components, especially in the case of transradial amputees (a more than 10% difference). Wilson et al. [18] even suggested that removal of the 20–120 Hz frequency band actually increased the classification accuracy of nine classes of motion significantly, and thus they recommend using the combination of sampling rate and high-pass cut-off frequency of 1000 Hz and 120 Hz to maximize classification performance in the presence of power line noise and motion artifacts. The present results agree with and extend upon our preliminary study [48] (which used a different EMG dataset of 20 able-bodied subjects, and considered another practical robustness issue, i.e., changes in the EMG signal itself over time) as well as those in [15,16,17].

The results of this investigation also included a comparison of twenty-six individual features (Table 3), which showed that the classification performance of *all* evaluated features decreased significantly (p<0.05) with the reduced sampling rate. It is interesting to note, however, that the signal amplitude and power features incurred less of a reduction than those in other feature groups. Other than the MFL feature, the effect size of the differences in the nonlinear complexity and frequency information features could be considered as *very large* or *huge* (*d* = 1.5–5.5). The drastic results found for this feature group may be explained by the loss of high-frequency content in the signal, and their corresponding power and complexity information, observed in both the time domain and frequency domain (Figure 1). In addition, although time-series modeling features (i.e., AR and CC) obtained good accuracies (89–91% on average) when using a 1000 Hz sampling rate, they suffered the largest decreases in accuracy when dropping to the 200 Hz sampling rate (over 35% on average; *d* = 7.5–8.8) (Table 3). This suggests that their benefit lies greatly in their use of the higher frequency content.

The changes in two EMG feature patterns were also examined visually to confirm the quantitative results (Figure 2). Based on two channels of EMG, ZC features extracted from EMG data sampled at 1000 Hz can mostly discriminate between thumb flexion and index flexion motions (Figure 2). These two classes, however, cannot be separated using the ZC features extracted from the same EMG data down-sampled to 200 Hz. This is demonstrated by the increased intra-class variability and consequent overlap of both motion classes.

Decreased classification performance of some features (such as AR, CC, DFA, and SampEn) could also be because of insufficient number of data points in each analysis window, i.e., a window size of 250 ms in this study contains 250 data points when using a 1000 Hz sampling rate but only 50 data points when using the 200 Hz sampling rate. For instance, Yentes et al. [54] suggested that SampEn is extremely sensitive to parameter choices in very short datasets (<200 data points) and recommended the number of data points to be larger than 200, and as large as possible with respect to the practical constraints of the application. Due to real-time constraints, however, the total response time for myoelectric control, which includes both the window size and processing delay, should not exceed 300 ms [2,37]. Consequently, algorithms that require longer windows of data may be not suitable for the new generation of low-sampling rate wearable EMG devices. In addition, several studies have computed EMG features with a smaller size of window (50–200 ms) than is used in this study, and showed acceptable classification accuracies (>90%) [2]. However, these studies investigated the dependence of features on window size in the classification of EMG signals sampled at 1000 Hz or above. Using a 200 Hz sampling rate, a window size of 50 ms contains only 10 data points, and a few data points would reduce not only the classification performance of features, but also the robustness of the systems. One should thus exercise caution when working on data segmentation (window size and window incrementation).

As a result of the relatively poor 200 Hz performance of features from all functional feature groups (with perhaps the exception of the signal amplitude and power feature group), a combination of features across these functional feature groups did not provide meaningful improvement in performance for the low-sampling rate myoelectric control systems. For example, MAV features yielded an overall classification rate of 76.4% for amputee subjects while the MS1 feature set consisting of MAV, WL, ZC and SSC yielded an overall classification rate of 78.4% for the same subjects (only a 2% increase). In contrast, same comparison yielded an overall increase of 9% when using the 1000 Hz sampling rate. Similarly, and importantly, the recruitment of more (or even all) features could not compensate for the loss of information due to the lower sampling rate in amputee subjects. In fact, as can be observed in Figure 3, the set of AR9 and MFL computed using data sampled at 1000 Hz drastically outperformed all combinations of 200 Hz features.

Another interesting and important finding is that the feature sets selected by the SFS algorithm were vastly different for the two sampling rates (Figure 3). Using 1000 Hz, the classification rate approached the first local maximum value when four features (AR9, MFL, MSR, and DASDV) were employed, and remained relatively constant when more features were added (i.e., no significant differences between consecutively selected added features). In contrast, when using 200 Hz, the first four features selected consisted of LS, MFL, VAR, and DVARV. The classification rates continuously increased and approached a maximum value when nine features were used, and decreased when more features were added. Only the MFL feature was common to the best sets of four features, and the differences between the two sets increased with the number of features included. These results suggest that previous feature selection works based on 1000 Hz sampling rates may not be applicable to lower sampling rate myoelectric control systems.

Losing useful high frequency control information becomes an even more serious problem for myoelectric control when considering how to reduce the training burden, i.e., when EMG data are acquired under one practical condition when training the classifier (Figure 4). It has been shown [24,25,26,27,28] that the performance of EMG classifiers drops significantly when tasked with identifying EMG patterns from unseen conditions. Consequently, advanced training protocols have been proposed as a way of introducing added variability during training [55]. These results are reinforced here as, for instance, using the MS1 feature set at 200 Hz, a 78.4% classification rate was found when training with data from all three contraction intensities (Table 2). When training with only two and one contraction intensities and testing with unseen contraction intensities, however, the corresponding classification rates dropped to 56.4% and 46.0%, respectively (Figure 4).

The second aim of the present study was to identify a set of recommended EMG features that provide better performance for myoelectric control based on low sampling rate wearable EMG sensors (here the Myo armband). This is the first study, to our knowledge, to experimentally perform a comprehensive investigation of the classification performance of a wide range of EMG features for myoelectric control based on this, or any other comparable, system. A similar trend was observed using the Myo (Figure 5) as when down-sampling the data collected from higher-sampling rate EMG devices (Table 3), for all tested features. It is important to note that, due to the low resolution of A/D converter used (8 bits), many data points in this dataset are zero-valued, and thus the LD feature cannot be computed (as the logarithm of zero is undefined; for a mathematical definition, see [12]). By adding a positive value of 1 to each data point, the feature could be computed, but the classification results obtained remained less accurate than other signal amplitude and power features. As the LD feature can be replaced by other features in the same group [32], we recommend to avoid using the LD feature with the Myo device.

As a standalone feature, the LS feature, proposed in this study, offers the highest classification rates (84.5% for Experiment A, 74.9% for Experiment B, and 69.1% for Experiment C), followed closely by the commonly used EMG amplitude estimators (i.e., IAV, MAV, RMS, and WL) and the more recently proposed features in the signal amplitude and power feature group (i.e., DAMV, DASDV, and MSR). As the newly proposed feature, LS, is less sensitive to outliers in data than conventional measures and yields a better classification performance, this novel feature is recommended to be used instead of the common features in this group. For other functional feature groups, MFL could be used to extract nonlinear and complexity information while WAMP could be used to extract frequency information in EMG signals.

In this study, the newly proposed multi-feature set TD4 selected using SFS consists of LS and MSR from the signal amplitude and power feature group and MFL and WAMP from the nonlinear complexity and frequency information feature group. This selection of features from complementary feature groups is consistent with our previous findings using topological data analysis [32].

The newly proposed EMG feature sets, TD4 and TD9, outperformed other state-of-the-art multi-feature sets developed based on EMG signals sampled at 1000 Hz or above (Figure 6). For instance, on average, the TD4 feature set improved classification accuracies by 1.8–3.4% (p<0.05) as compared to the most commonly used Hudgins’ TD feature set (MS1) with the same number of features. Furthermore, on average, the TD9 feature set improved classification accuracies by 3.5–5.5% (p<0.05) as compared to the best of the eight state-of-the-art multi-feature sets (MS3), with a lower feature vector dimension. Therefore, these two newly proposed feature sets are recommended to be used for myoelectric control based on the new generation of lower-sampling rate wearable EMG sensors.

### Limitations and Future Studies

The advent of non-invasive wearable EMG bands yields the promise of improved and more convenient human–computer interfaces for amputees and able-bodied individuals alike. Nevertheless, to improve their practicality and reliability, further studies are required that examine the effects of dynamic and confounding factors such as changes in EMG signal over time, limb position, forearm orientation, contraction intensity, and electrode locations. Other related factors in surface EMG measurement including but not limited to resolution of A/D, types of electrode, impedance, and cross talk, could be explored to better understand the performance of EMG feature extraction and pattern recognition techniques in emerging wearable EMG technology.

The current findings clearly showed that high-frequency content (>100 Hz) is critical to the performance of myoelectric control system. New and emerging EMG armbands should therefore be developed with due consideration for the trade-offs between design considerations such as power consumption and memory, and performance, such as accuracy and robustness. Further research investigating the wide range of potential sampling issues of EMG signals in motion recognition is required.

Finally, in this contribution, we focused on surface EMG signals. However, the loss of high frequency components could also degrade the classification of similar types of feature extraction methods in other biological signals used as prosthetic control signals [56]. The methods described here could be applied to evaluate other types of biological signals for a better understanding of the sampling rate effect in other areas of prosthetic control.

## 5. Conclusions

This investigation clearly showed that sampling rate has a significant impact on classification performance in identifying different classes of hand and finger movements. Using a 200 Hz sampling rate, as is used in the predominant commercially available wearable EMG armband, instead of a 1000 Hz sampling rate, results in a drastic reduction in discriminative information for use in myoelectric control. We further present two purposely engineered sets of EMG features, TD4 and TD9, including a novel feature, LS, for use in myoelectric control using lower-bandwidth wearable EMG sensors.

## Figures and Tables

**Table 1:** Four EMG datasets: subjects, experiment, and data acquisition.

	Dataset 1 [26]	Dataset 2 [27]	Dataset 3 [28]	Dataset 4 [31]
Practical Issue(s)	Limb position (5 positions)	Forearm orientation (3 orientations), Contraction intensity (3 levels)	Amputation, Contraction intensity (3 levels)	Wearable sensors (Myo armband)
Number of Subjects	11 (able-bodied)	10 (able-bodied)	9 (amputee)	10 (able-bodied)
Number of Movements	7	6	6	52 (12 + 17 + 23)
Number of Repetitions	6	3	5–11	6
Total Number of Trials	2310	1620	1077	3120
Time for Each Trial	5 s	5 s	8–12 s	5 s
Original Sampling Rate	4000 Hz	4000 Hz	2000 Hz	200 Hz
Resolution	12-bit	12-bit	16-bit	8-bit
Number of Electrodes	7	6	8	16

**Table 2:** Classification performance of 8 multi-feature sets using an SVM classifier when the training and testing sets are acquired from multiple conditions for multiple EMG datasets (Datasets 1–3).

Multi-Feature Set	Able-Bodied Subjects									Amputee Subjects			Mean		
	Limb Position			Forearm Orientation			Contraction Intensity			Contraction Intensity					
	1000	200	*d*	1000	200	*d*	1000	200	*d*	1000	200	*d*	1000	200	*d*
MS1 (TD)	98.3	93.9	1.7 *	98.2	92.7	1.3 *	99.1	96.8	1.0 *	89.8	78.4	1.6 *	96.3	90.4	1.4
MS2	98.8	93.3	2.0 *	98.7	92.0	1.5 *	99.3	96.2	1.2 *	91.9	78.7	2.1 *	97.2	90.1	1.7
MS3	99.1	94.1	2.1 *	99.1	92.4	1.6 *	99.5	96.7	1.2 *	93.5	79.9	2.2 *	97.8	90.8	1.8
MS4	97.1	84.6	3.5 *	96.9	84.8	2.2 *	97.7	88.6	1.8 *	88.5	72.1	2.2 *	95.0	82.5	2.4
MS5	98.9	93.4	2.1 *	98.7	91.6	1.6 *	99.4	95.6	1.3 *	92.7	77.6	2.4 *	97.4	89.6	1.8
MS6	98.6	92.6	2.1 *	98.1	89.6	1.9 *	99.0	94.0	1.5 *	91.9	76.1	2.6 *	96.9	88.1	2.0
MS7	97.1	93.8	1.2 *	97.6	92.8	1.2 *	98.6	96.2	0.9 *	85.9	78.8	0.9 *	94.8	90.4	1.0
MS8	99.1	93.7	2.1 *	99.0	92.2	1.6 *	99.6	96.4	1.4 *	93.1	79.6	2.1 *	97.7	90.5	1.8

* Denotes a significant difference between 1000 Hz and 200 Hz (p<0.05).

**Table 3:** Classification performance of 26 individual features using an SVM classifier when the training and testing sets are acquired from multiple conditions for multiple EMG datasets (Datasets 1–3).

Feature Set	Able-Bodied Subjects									Amputee Subjects			Mean		
	Limb Position			Forearm Orientation			Contraction Intensity			Contraction Intensity					
	1000	200	*d*	1000	200	*d*	1000	200	*d*	1000	200	*d*	1000	200	*d*
IAV	95.2	92.6	0.7 *	94.9	91.4	0.6 *	98.4	96.3	0.7 *	80.8	76.4	0.6 *	92.3	89.2	0.7
MAV	95.2	92.6	0.7 *	94.9	91.4	0.6 *	98.4	96.3	0.7 *	80.8	76.4	0.6 *	92.3	89.2	0.7
RMS	95.2	92.5	0.7 *	94.8	91.1	0.6 *	98.2	96.2	0.7 *	80.7	76.8	0.5 *	92.2	89.2	0.6
VAR	93.1	89.2	0.7 *	90.3	86.7	0.5 *	94.7	92.9	0.3 *	75.5	70.7	0.5 *	88.4	84.9	0.5
WL	95.9	92.7	0.9 *	96.2	91.1	0.9 *	98.4	96.5	0.7 *	80.4	75.2	0.6 *	92.7	88.9	0.8
DAMV	95.9	92.7	0.9 *	96.2	91.1	0.9 *	98.4	96.5	0.7 *	80.4	75.2	0.6 *	92.7	88.9	0.8
DASDV	95.9	92.7	0.9 *	95.9	91.1	0.9 *	98.3	96.2	0.7 *	79.8	75.5	0.5 *	92.5	88.9	0.8
DVARV	93.6	89.5	0.7 *	90.7	86.5	0.6 *	94.6	92.6	0.4 *	75.5	70.0	0.6 *	88.6	84.7	0.6
LS	95.3	92.6	0.7 *	95.0	91.5	0.6 *	98.4	96.4	0.7 *	81.1	77.0	0.6 *	92.4	89.4	0.6
MSR	95.4	92.3	0.9 *	95.5	91.7	0.7 *	98.4	96.4	0.7 *	80.7	75.1	0.7 *	92.5	88.9	0.7
LD	94.4	90.2	1.0 *	94.0	89.7	0.7 *	97.8	94.7	0.9 *	76.9	69.6	0.9 *	90.7	86.1	0.9
MFL	96.5	93.1	1.1 *	97.2	92.1	1.0 *	98.9	96.8	0.8 *	81.2	76.1	0.6 *	93.4	89.5	0.9
DFA	72.0	32.4	9.5 *	63.3	33.8	4.6 *	69.6	36.2	3.2 *	58.4	27.9	4.6 *	65.8	32.6	5.5
SampEn	72.9	16.7	12.0 *	66.4	18.3	7.9 *	70.1	19.3	7.3 *	56.9	17.9	5.4 *	66.6	18.1	8.1
ZC	66.2	54.3	2.1 *	58.0	54.1	0.5	68.0	59.7	0.8 *	53.6	34.5	2.6 *	61.4	50.6	1.5
SSC	66.2	38.4	5.4 *	60.2	39.6	3.1 *	64.1	43.6	2.7 *	53.6	31.0	2.7 *	61.0	38.1	3.5
WAMP	92.0	79.0	2.7 *	91.9	77.7	1.9 *	96.1	81.5	2.6 *	61.5	49.2	1.2 *	85.4	71.8	2.1
MDF	72.4	44.0	5.5 *	64.2	45.5	2.6 *	72.9	49.9	2.1 *	56.2	35.0	3.2 *	66.4	43.6	3.4
MNF	77.5	50.2	5.2 *	71.1	51.1	2.8 *	77.4	55.9	2.0 *	65.3	38.7	3.8 *	72.8	48.9	3.4
AR4	93.6	54.4	11.2 *	92.2	54.1	8.2 *	94.2	58.9	6.9 *	81.8	45.3	5.7 *	90.5	53.2	8.0
AR6	94.3	54.5	11.7 *	93.2	54.0	8.4 *	95.1	58.4	8.2 *	83.1	45.4	6.1 *	91.4	53.1	8.6
AR9	94.5	54.8	10.8 *	93.2	54.0	8.7 *	95.5	58.6	9.3 *	83.9	45.2	6.1 *	91.8	53.2	8.8
CC4	93.4	54.3	10.7 *	91.2	54.0	7.4 *	93.2	58.6	6.1 *	81.3	45.0	5.8 *	89.8	53.0	7.5
CC6	94.0	54.6	10.7 *	91.6	54.0	7.4 *	94.1	58.2	6.1 *	82.3	45.4	5.8 *	90.5	53.0	7.5
CC9	94.1	54.8	10.4 *	91.5	53.4	8.0 *	94.3	57.5	8.4 *	83.4	44.8	6.4 *	90.8	52.6	8.3
HIST9	84.9	80.2	0.8 *	84.2	81.3	0.4	88.7	85.8	0.3	72.1	68.0	0.5 *	82.5	78.8	0.5

* Denotes a significant difference between 1000 Hz and 200 Hz (p<0.05).
